# The Value of Negative-Pressure Wound Therapy and Flap Surgery in Hidradenitis Suppurativa – A Single Center Analysis of Different Treatment Options

**DOI:** 10.3389/fsurg.2022.867487

**Published:** 2022-06-28

**Authors:** M. C. Stumpfe, R. E. Horch, A. Arkudas, A. Cai, W. Müller-Seubert, T. Hauck, I. Ludolph

**Affiliations:** Department of Plastic and Hand Surgery, Laboratory for Tissue Engineering and Regenerative Medicine, University Hospital Erlangen, Friedrich-Alexander University Erlangen-Nürnberg (FAU), Erlangen, Germany

**Keywords:** negative-pressure wound therapy, acne inversa, hidradenitis suppurativa, bacterial burden, flap surgery

## Abstract

**Background:**

Hidradenitis suppurativa is manifested by painful abscesses and scarring of sweat glands. Axillary, inguinal and genital regions are mostly affected. Multiple options exist in the treatment of hidradenitis suppurativa. The aim of this retrospective, mono-center cohort study was to analyze the outcome of different treatment methods after radical excision of hidradenitis suppurativa.

**Methods:**

We retrospectively evaluated the treatment strategy and recurrence rate of hidradenitis suppurativa. We included all eligible patients of legal age between February 2003 and October 2021, with the diagnosis of Hidradenitis suppurativa and the necessity for surgical treatment. All patients with surgical treatment and direct wound closure by suture were excluded. Bacterial load and flora were analyzed for primary and secondary reconstruction in combination with negative-pressure wound therapy. Patient data were analyzed for recurrence rate and remission time according to different reconstructive techniques.

**Results:**

In 44 affected anatomical sites (*n* = 23 patients) we treated 15 patients with negative-pressure wound therapy. Bacterial load and flora were lower in the last wound swab of patients with multi-surgical procedures (22 localizations) compared to the first wound swab independent of the use of negative-pressure wound therapy.

Wound closure, independent of a direct and multi-stage procedure was achieved by local fasciocutaneous flaps (*n* = 12), secondary intention healing (*n* = 7), secondary intention healing with buried chip skin grafts (*n* = 10), or split**-**thickness skin grafts (*n* = 15). Radical excision combined with split**-**thickness skin grafts showed the lowest recurrence rate in the follow-up (16%; *n* = 4).

**Conclusion:**

Radical excision of hidradenitis suppurativa as gold standard for surgical treatment combined with negative-pressure wound therapy as multi-stage procedures ultimately reduced bacterial load and flora in our study. The use of split**-**thickness skin grafts showed the lowest recurrence rate.

## Introduction

Hidradenitis suppurativa (HS), also known as acne inversa, is a painful disease with a relapsing-remitting character (1, 2). First described by Velpeau in 1839, Verneuil related the hidradenitis suppurativa to sweat glands in 1854 (1). Axillary, inguinal, and genital regions are mostly affected (3). Other regions with apocrine glands can be partially afflicted, including e.g. the breast areola or the submammary zone (3–5). The first manifestations usually occur in adolescence (1).

Hidradenitis suppurativa is manifested by painful abscesses and scarring (1). The severity of hidradenitis suppurativa is classified according to the Hurley classification defining three different stages (Table 1) (2, 6).

**Table 1:** The Hurley classification of hidradenitis suppurativa.

Stage 1	Single or multiple abscess formation without sinus tracts and cicatrization
Stage 2	Recurrent single or multiple abscesses, widely separated, with limited sinus tracts and cicatrization
Stage 3	Diffuse or near-diffuse involvement of multiple interconnected tracts and abscesses across an entire area

Endocrine abnormalities and bacterial infection can be associated with the pathogenesis (7, 8). Staphylococcus aureus and Staphylococcus epidermidis are common pathogenic bacteria found in the context of this disease (9). Additionally, obesity, smoking, and genetic predisposition are common risk factors (1, 7, 8).

Radical surgical excision remains the gold standard for the treatment of HS in stage 2 and 3 according to the Hurley classification (2).

There are various therapeutic options of HS, but there is no common consensus concerning wound treatment following excision (10). Traditionally one would suppose that a solid soft tissue flap cover would provide the best long-term coverage, but the authors experienced superior results with split-thickness skin grafts in terms of lesser recurrences over time.

In this study, we compare different treatment strategies and reconstructive options after radical surgical excision including the use of negative-pressure wound therapy (NPWT).

## Methods

**Figure 1:**
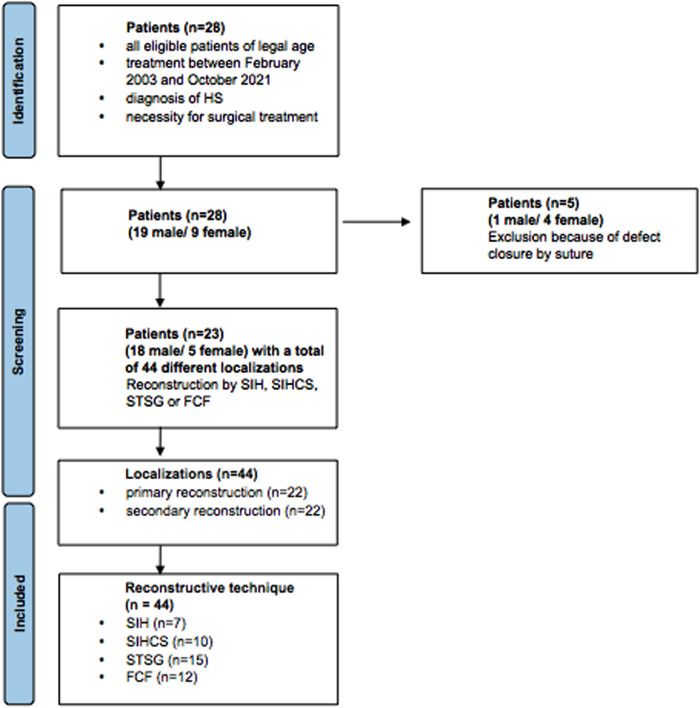
Flow chart with included patient numbers and reasons for exclusion and illustration of the subgroups primary vs. secondary reconstruction and reconstructive technique; SIH, secondary intention healing; SIHCS, secondary intention healing with buried chip skin grafts; STSG, split-thickness skin graft; FCF, fasciocutaneous flaps.

In a single-center study, we retrospectively analyzed data of patients treated for HS. We included all eligible patients of legal age between February 2003 and October 2021, with the diagnosis of HS and the necessity for surgical treatment. All patients underwent a radical surgical debridement until all macroscopically affected pathological tissue was removed. All patients who were treated conservatively or by simple suture were excluded from the study (Figure 1).

We subdivided patients into cases and affected localizations. Admission of a patient to the hospital due to a previously untreated localization was counted as a new case. Multiple affected localizations treated in a single patient during the same hospital stay were summarized as one case.

Furthermore, cases were classified into primary reconstruction (PR) and secondary (multi-stage/SR) reconstruction. For primary reconstruction primary wound closure (PC), secondary intention healing with or without buried chips skin grafts (SIH, SIHCS), pedicled fasciocutaneous flaps (FCF), or split-thickness skin grafts (STSG) were performed (11–13).

The multi-stage collective was divided into the no-NPWT, NPWT, and NPWT with instillation and dwell time (NPWTi-d) group. Wound closure was achieved by secondary closure (SC), pedicled fasciocutaneous flaps (FCF), secondary intention healing with buried chip skin grafts (SIHCS), or split**-**thickness skin grafts (STSG).

The patients with no-NPWT/NPWT/NPWTi-d received multiple surgical debridements and, if applied, regular changes of the negative-pressure wound dressing (3M Deutschland Gmbh. Neuss, Germany). As an antiseptic solution, Polyhexanide (0.4 mg/1 mL) (Lavasept, B. Braun Medical AG, Germany) was used for NPWTi-d. The instillation volume was individually correlated to the defect size. Dwell time was set to 20 min. During hospitalization reconstruction of the affected anatomical site was performed by split**-**thickness skin grafts, buried chip skin grafts or, pedicled fasciocutaneous flaps. Following wound closure by STSG, patients received additional NPWT on top of the STSG until the fifth day without any dressing changes meanwhile. The negative pressure was adjusted to −125 mmHg in a continuous mode.

Intravenous antibiotics were administered following defect coverage according to the antibiograms of the actual wound swabs.

Outpatient records were screened with special attention to the recurrence of HS. The time between surgery and recurrence with a necessity for surgical treatment was noted. Patients without re-presentation in our clinic were counted as recurrence-free.

Due to the small subgroups, no distinction between PR and SR regarding recurrence rate was made.

Additionally, we analyzed the results of the first and the last available wound swab taken during surgical procedures and the bacterial flora (number of different bacterial species) and load (amount of bacteria under culture) between different treatment groups including NPWT/NPWTi-d. The number of different bacterial species (NDB/ flora) was analyzed by the local Institute for Clinical Microbiology by the differentiation of microbial types, including all kinds of germs, existing in the wound. The amount of bacteria under culture (AB/load) was measured semi-quantitative based on the rough data of the microbiological analysis of the cultural cultivation of the wound swab according to the routine procedure of the local Institute for Clinical Microbiology. This assessment allows a rough estimation of the severity of bacterial colonization with a pseudo-numerical classification including “after enrichment “(0.5), “sparse” (1), “moderate” (2), “several” (3), or “plenty” (4) and is commonly used in routine clinical practice (Table 2).

**Table 2:** Overview of the quantity of bacteria and semi-quantitative description of bacteria.

Quantity of bacteria under culture	Semi-quantitative description
After enrichment	0.5
Sparse (+)	1.0
Moderate (++)	2.0
Several (+++)	3.0
Plenty (++++)	4.0

All relevant data according to the criteria were analyzed using Excel (Microsoft, Redmond, Washington, USA).

The primary surrogate parameter was the recurrence rate following surgical treatment. Further results included the change of bacterial load and flora in cases with secondary reconstruction.

The retrospective and anonymous character of this study is in accordance with the institutional ethics committee and the Helsinki Declaration and its later amendments or comparable ethical standards.

### Statistical Analysis

We analyzed patient demographics by descriptive statistics. For subgroup analysis, we also performed descriptive statistics due to the small group data and the expectable low statistical power.

Analyses were performed using GraphPad Prism Version 6 (GraphPad Software, Inc., California, USA).

## Results

### Total Patient Collective Demographics

A total of 28 patients (19 male, 9 female) were analyzed. Five patients were excluded because of primary or secondary closure by suture after wound conditioning with or without NPWT/NPWTi-d. Therefore, 23 patients (18 male, 5 female) were included in the study with 35 cases of hidradenitis suppurativa. In total, 44 wound localizations were treated. The age of the patients ranged from 14.4 to 55.8 years (mean age 33.9 years). The included patients suffered from HS between 0.1 and 40.7 years (mean 10.2 years). All patients received multiple local incisions in the past without radical debridement and reconstruction. Three (13.0%) patients changed from a primary to a secondary reconstruction when another localization was affected or recurrence occurred and a single-stage treatment was not promising.

### Wound Characteristics

#### Primary Reconstruction vs. Secondary Reconstruction

There was no difference between the two groups in terms of gender distribution, age, and disease duration. Patients with a secondary reconstruction had a mean duration between debridement and reconstruction of 20.3 days (range 3.0–56.0 days). The maximum span of 56 days between debridement and treatment was necessary for a multi-morbid patient who presented cardiopulmonary complications during the hospital stay leading to a delay in the operative treatment. Meanwhile, the patient was discharged from the hospital until cardiopulmonary recompensation. Details are listed in Table 3.

**Table 3:** Demographic data, risk factors and localization of the primary and secondary treated patients.

Characteristic	Primary (*n* = 22)	Secondary (*n* = 22)
Female gender	36% (8/22)	22% (5/22)
Mean age in years (SD)	34.0 ± 10.9	39.7 ± 12.8
Disease duration in years^a^ (mean and range)	8.7 (0.1–40.7)	13.3 (0.2–40.7)
Mean length (range min – max) of hospital stay in days	11.5 (4.0–24.0)	18.7 (7.0–36.0)
Risk factors		
Smoking	41% (9/22)	18% (4/22)
Obesity	5% (1/22)	36% (8/22)
Diabetes mellitus 2	0% (0/22)	9% (2/22)
Localization		
Axilla right	4	3
Axilla left	3	7
Inguinal right	5	4
Inguinal left	4	4
Anogenital	4	3
mons pubis	2	1
Median duration (range min – max) between debridement and reconstruction in days	n/a	20.3 (3.0–56.0)

^a^ *Until intervention; primary, primary reconstruction; secondary, secondary reconstruction; n, number of localizations.*

#### Reconstructive Technique

Twenty-seven (61.4%) of 44 affected regions were treated with STSG (*n* = 15) or FCF (*n* = 12). The axillary region treated with STSG or FCF was the most affected anatomical site (*n* = 16, 59.3%). In 53.0% (*n* = 8) of the STSG a NPWT/NPWTi-d before reconstruction was applied. Ten localizations (22.7%) were treated by buried chip skin grafts in the inguinal or anogenital region. Details regarding reconstructive techniques are recorded in Table 4.

**Table 4:** Overview of the reconstruction techniques distributed among the localizations; Categorical data are expressed as % (n/N).

Characteristic	SIH (*n* = 7)	SIHCS (*n* = 10)	STSG (*n* = 15)	FCF (*n* = 12)
Female gender	14% (1/7)	0% (0/10)	46% (7/15)	42% (5/12)
Mean age	31.2 ± 11.4	47.0 ± 5.6	33.7 ± 13.8	37.4 ± 8.9
Disease duration in years^a^ (mean and range)	11.5 (4.3–40.7)	18.7 (4.1–28.5)	13.7 (1.9–40.7)	8.8 (0.8–20.3)
Mean length (range min – max) of hospital stay in days	10.1 (5.0–15.0)	19.6 (7.0–36.0)	16.2 (6.0–32.0)	13.8 (4.0–29.0)
Risk factors				
Smoking	43% (3/7)	40% (4/10)	13% (2/15)	33% (4/12)
Obesity	14% (1/7)	0% (0/10)	46% (7/15)	8% (1/12)
Diabetes mellitus 2	0% (0/4)	0% (0/10)	7% (1/15)	8% (1/12)
Localization				
Axilla right	1	0	4	2
Axilla left	0	0	7	3
Inguinal right	2	3	2	2
Inguinal left	1	3	2	2
Anogenital	2	4	0	1
Mons pubis	1	0	0	2
Mean duration between debridement and reconstruction in days	n/a	34.0 (13.0–56.0)	14.7 (3.0–27.0)	11.8 (6.0–21.0)
No-NPWT/NPWT/NPWTi-d	7/0/0	7/3/0	7/4/4	8/2/2

*Continuous data are expressed as mean ± SD.*

*SIH, secondary intention healing; SIHCS, secondary intention healing with buried chip skin grafts; STSG, split-thickness skin graft; FCF , fasciocutaneous flaps, no-NPWT, no negative-pressure wound therapy, NPWT, negative-pressure wound therapy; NPWTi-d, negative-pressure wound therapy with instillation and dwell time.*

^a^ *Until intervention.*

### Wound Healing Outcome

Patients were in remission for a mean of 29.8 months (range 3.0–84.0 months) regardless of the reconstructive technique used. STSG showed the lowest recurrence rate and the longest disease-free period compared to all other reconstructive techniques.

**Figure 2:**
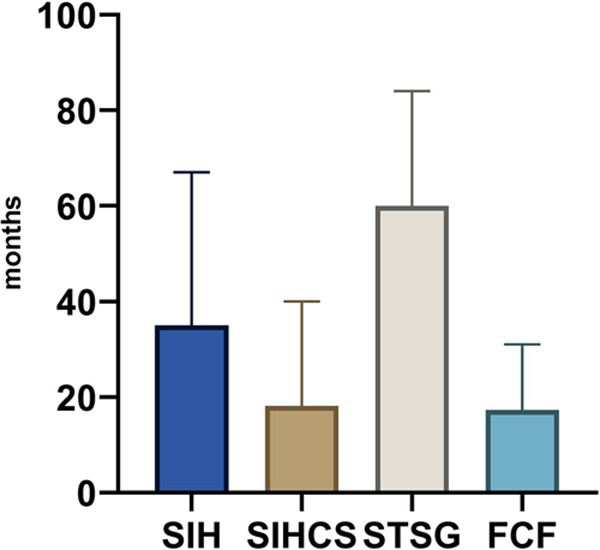
Graphical representation of the time until recurrence in months between reconstruction and recurrence (mean); SIH, secondary intention healing; SIHCS, secondary intention healing with buried chip skin grafts; STSG, split-thickness skin graft; FCF, fasciocutaneous flaps; PR, primary reconstruction; SR, secondary reconstruction.

Patients who received split-thickness skin grafts were disease-free at the treated site for a mean of 60 months (range 5–84 months). Further details can be taken from Figure 2 and Table 5.

**Table 5:** Detailed evaluation of the recurrence rate and time until recurrence in months between reconstruction and recurrence (mean).

Characteristic	SIH (*n* = 7)	SIHCS (*n* = 10)	STSG (*n* = 15)	FCF (*n* = 12)
Recurrences	29% (2/7)	60% (6/10)	26% (4/15)	33% (4/12)
Mean duration (range) between reconstruction and recurrence in months	35.0 (3.0–67.0)	18.1 (1.0–40.0)	60.0 (5.0–84.0)	17.3 (8.0–31.0)

*SIH, secondary intention healing; SIHCS, secondary intention healing with buried chip skin grafts; STSG, split-thickness skin graft; FCF, fasciocutaneous flaps.*

We divided postoperative complications according to the Clavien–Dindo classification. In total, nine (28.0%) postoperative complications occurred. Grade I (*n* = 6) included wound dehiscence (*n* = 4) and mild scar contracture (*n* = 2) treated conservatively. Reoperation according to Grade IIIb (*n* = 3) was required in the FCF group because of a postoperative hematoma (*n* = 2) and a perfusion problem in one case of FCF, which was treated by revision of the FCF. None of these cases showed further complications and no flap loss occurred.

### Bacterial Load and Flora

A total of 26 different bacteria were detected by wound swabs. Most frequently, *saprophytic gram-positive bacteria* (*n* = 11)*, mixed cultures of anaerobic bacteria* (*n* = 5), and *Streptococcus anginosus* (*n* = 4) were detected.

Overall, wound swab analysis showed aerobic bacteria in 54.0% (*n* = 27) and gram-positive bacteria in 76% of the wound swabs (*n* = 37).

Two patients presented with severely infected wounds by *methicillin-resistant staphylococcus aureus (MRSA)*. In both cases, bacteria could be detected also in the last wound swab before wound closure. One patient received an FCF in the anogenital region after radical debridement. No postoperative complications occurred. Recurrence was diagnosed 8 months postoperatively. In the second case, STSG in the axilla region was performed after therapy using NPWTi-d. Both, postoperative complications and recurrence were not reported.

#### Primary Reconstruction

The average number of different bacteria (NDB) for PR (*n* = 22) was 1.9 (SD 1.6; range 0.0–5.0). The mean amount of bacteria (AB) was 2.7 (SD 1.8; range 0.0–5.0).

#### Secondary Reconstruction

The average NDB for all localizations in the subgroup secondary reconstruction (*n* = 22) before reconstruction was 0.9 (SD 0.8; range 0.0–2.0) and the AB 1.7 (SD 2.0; range 0.0–6.0)

**Figure 3:**
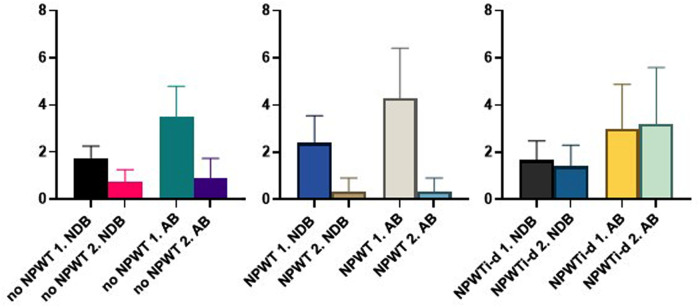
Development of the bacterial flora (NDB) and load (AB) (before debridement (1. NDB/1. AB) and before reconstruction (2. NDB/2.AB); NPWT, negative pressure wound therapy; NPWTi-d, negative-pressure wound therapy with instillation and dwell time.

The first wound swab before debridement of patients with no NPWT revealed a mean NDB bacteria of 1.8 (SD 0.5; range 1.0–2.0) and AB of 3.5 (SD 1.3; range 2.0–5.0). Patients with NPWT had an NDB of 2.4 (SD 1.1; range 1.0–4.0) and an AB of 4.3 (SD 1.9; range 2.0–7.5) in the first wound swab. For patients treated with NPWTi-d NDB was 1.7 (SD 0.8; range 1.0–3.0) and AB 3.0 (SD 1.8; range 0.5–6.0) in the first wound swab. For the last wound swab before wound coverage, the results of the NDB and the AB are summarized in Table 6. The development of the bacterial burden is illustrated in Figure 3.

**Table 6:** Summary of the wound swabs results; bacterial load (AB) and flora (NDB) before debridement (1. NDB/ 1. AB) and immediately before reconstruction (2. NDB; 2. AB).

	no NPWT				NPWT				NPWTi-d			
	1. NDB	2. NDB	1. AB	2. AB	1. NDB	2. NDB	1. AB	2. AB	1. NDB	2. NDB	1. AB	2. AB
Mean	1.8	0.8	3.5	0.9	2.4	0.3	4.3	0.3	1.7	1.4	3.0	3.2
Minimum	1.0	0.0	2.0	0.0	1.0	0.0	2.0	0.0	1.0	0.0	0.5	0.0
Maximum	2.0	1.0	5.0	2.0	4.0	1.0	7.5	1.0	3.0	2.0	6.0	6.0
SD	0.5	0.5	1.3	0.85	1.1	0.6	2.1	0.6	0.8	0.9	1.9	2.4

*NPWT, negative pressure wound therapy; NPWTi-d, negative-pressure wound therapy with instillation and dwell time.*

In four localizations (26.6%) of NPWT/NPWTi-d no bacteria was found in the last wound swab.

## Discussion

The treatment options of hidradenitis suppurativa are wide-ranging. The conservative treatment options are diverse and include antibiotic or hormone therapy, steroids, and treatment with biologicals (14). In many cases, conservative treatment is not successful and leads to a chronic and/or progressive course of the disease.

The gold standard for surgical therapy is a radical excision of the affected areas. It has the highest healing potential and less recurrence rates compared to local incisions or deroofing interventions (15, 16). The value of topical negative-pressure wound therapy, especially with an additional instillation of antiseptics has been described several times and is intended to minimize surgical site infections and optimize the final reconstructive results (17–23).

However, there is yet no general consensus for the optimal reconstructive procedure following surgical therapy. The choice of the reconstructive technique depends on the dimension of the defect as well as the affected area (1). In this study, the patient population was retrospectively reviewed and compared by subgroup analysis concerning the reconstructive procedure, the use of negative-pressure wound therapy, and bacterial wound burden and recurrence rate amongst others. Other than in irradiated sites where flaps are a prerequisite for permanent closure, skin grafts do have an important place in the treatment of HS (24–27)

Many patients suffer from a chronic and exhausting course including ineffective therapy efforts with multiple interventions. Single minor lesions can be treated by direct excision and suturing. However, direct wound closure is usually not possible due to defect size and inflammation. For the latter ones, a high degree of surgical expertise is necessary and therefore requires the treatment in an experienced center.

Tissue engineering, especially generation of skin substitutes as well as later methods of regenerative medicine to replace the lost soft or hard tissue and/or skin is a promising new tool in this context (28–32).

However, current research has not yet reached the stage where soft tissue defects can be treated by tissue engineering (33, 34).

Our data show that radical excision combined with split skin grafting following wound bed preparation with or without NPWT/NPWTi-d descriptively had the longest relapse free remission duration. The presented results are consistent with the literature. This underlines the notes that defect coverage with meshed split**-**thickness skin grafts has the lowest rate of recurrence (6%) compared to local fasciocutaneous flaps (8%) or excision with simple direct wound closure (15%) (15, 16, 35). A meta-analysis by Ovadja et al. found that direct wound closure in terms of a primary suture had the worst outcome related to the development of recurrence (35). In their study defect reconstruction with meshed split**-**thickness skin grafts or local fasciocutaneous flaps has been described to have similar outcomes (2% each) (35). They also noted that Hidradenitis suppurativa below the umbilicus was significantly associated with higher overall recurrence (35). However as Ovadja et al. stated, the quality of evidence of their literature review was poor, and the reporting of result was mostly heterogeneous (35).

In our study, NPWT/NPWTi-d was primarily intended to reduce the bacterial burden while at the same time wound bed preparation before reconstruction was achieved. The comparison between primary and multi-stage wound closure showed a lower number of different bacteria as well as a decrease of the amount of bacteria following wound bed preparation. The results of this study underline that multi-stage therapy can improve the bacterial wound burden. NPWT/NPWTi-d has the potential to decrease bacterial load and flora in HS and therefore facilitates wound healing outcomes as known from different indications and localizations (17, 36, 37). According to data regarding NPWTi-d, the second wound swab shows a slight increase in AB. In the detailed evaluation, this can be explained by the MRSA colonization of one patient. Overall, there was a reduction in NDB but an increase in AB most likely due to MRSA. The effect of NPWTi-d on multidrug-resistant bacteria needs to be investigated in future studies.

The use of NPWT/NPWTi-d is known to lead to a beneficial blood flow alteration across the wound bed (20). This leads to various effects on a macroscopic and microscopic level (38). NPWT/NPWTi-d prepares wounds for closure, protects granulation tissue, and reduces the risk of wound infections (39, 40). Macrodeformation leads to a reduction of interstitial fluid-like hematoma and seroma and opens capillaries close to the wound bed. It is supposed to improve perfusion of a wound, increase the relief of growth factors and so support cellular progression (20). To the best of our knowledge still today no data are available for the use of NPWT/NPWTi-d compared to different treatment strategies in HS.

There are case reports and case series which reported a benefit of the use of NPWT/NPWTi-d for the treatment of HS (39–42). A secondary reconstruction after radical excision is a well-established strategy compared to a single-stage design (43). Patients with secondary reconstruction had better postoperative outcomes. In this group of patients according to our study, the bacterial load and flora were reduced by NPWT/NPWTi-d. In addition, this group showed fewer wound healing disorders in the postoperative course.

An additional benefit of NPWT/NPWTi-d is that daily dressing changes are not necessary. The dressing can be left in place for several days. Thus, affected patients experience less pain and greater treatment comfort.

This study is limited by its retrospective design and the small subgroups. Additionally, there is no consistent definition of disease recurrence in the literature. Prospective studies including larger subgroups are necessary to analyze different treatment strategies after radical debridement. Furthermore, the effect of NPWT/NPWTi-d on multidrug-resistant bacteria must be investigated in prospective studies.

Despite its retrospective design, this study presents the importance of further elucidating therapeutic options and underlines the strategy of a radical debridement with wound conditioning before reconstruction.

## Conclusion

The therapy of hidradenitis suppurativa continues to be in the focus of clinical and experimental studies.

Radical excision of hidradenitis suppurativa is the gold standard for surgical treatment. The use of negative pressure wound therapy shows a high potential in ultimately reducing bacterial load and flora. Split**-**thickness skin grafts showed the fewest complications in this study and presented the longest remission.

## Data Availability

The raw data supporting the conclusions of this article will be made available by the authors, without undue reservation.
